# The Clinical Burden of Rotavirus Gastroenteritis: A Prospective Study

**DOI:** 10.7759/cureus.1903

**Published:** 2017-12-02

**Authors:** Ahmed Nahari, Salem M AlGhamdi, Abdulsalam Alawfi, Hassan Faqeehi, Saeed Alzahrani, Amani Abu-Shaheen, Abdulrahman Al-Hussaini

**Affiliations:** 1 Pediatrics, King Fahad Central Hospital, Jazan, Saudi Arabia; 2 Division of Pediatric Infectious Disease, King Fahad Medical City; 3 Pediatrics, Taibah University; 4 Children Specialized Hospital, King Fahad Medical City; 5 Pediatrics, King Abdullah Bin Abdulaziz University Hospital, Riyadh; 6 Research Center, King Fahad Medical City; 7 Division of Gastroenterology, Children’s Specialized Hospital, King Fahad Medical City, College of Medicine at Alfaisal University, Riyadh, Saudi Arabia

**Keywords:** rotavirus infection, rotavirus gastroenteritis, saudi arabia.

## Abstract

Background

In Saudi Arabia, there is a lack of recently published, appropriately conducted epidemiological studies on rotavirus (RV) diarrhea, which emphasizes the need for up-to-date and comprehensive studies.

Objective

Our objective was to provide more recent data on the clinical and epidemiological characteristics as well as the economic burden of RV diarrhea among young children admitted to a tertiary care hospital in the city of Riyadh in the year prior to the initiation of the RV vaccine.

Design

We conducted a prospective observational study at a children’s specialized hospital at King Fahad Medical City. We included children under five years of age who were hospitalized for gastroenteritis over a 12-month period from January 2012 to December 2012. Stool samples were collected on admission and tested for the presence of RV using an enzyme immunoassay.

Results

Of the 204 children included over the study period (mean age, 9.8 months ± 10.2; 124 males), 102 (50%) were RV-positive. Two-thirds (69.6%) were under one year old, and 38.2% were under six months of age. RV infections occurred throughout the year, with the highest proportion occurring during the spring and summer. RV-positive diarrhea was more severe than the RV-negative diarrhea as indicated by a significantly lower bicarbonate level (68.6% versus 31.3%, P-value < 0.0001), a higher frequency of severe dehydration (11.7% versus 3%, P-value = 0.015), and longer hospital stay (mean duration, 8.78 versus 6.56 days, P-value = 0.027). In addition, the financial burden of the RV-positive cases was greater than the RV-negative cases (median 1692 USD versus 1287 USD, P-value = 0.001).

Conclusion

Our study shows a high prevalence of RV infections among young children admitted to the hospital for acute gastroenteritis. Furthermore, RV infections are associated with severe diarrhea and significant financial burden.

## Introduction

Rotavirus (RV) affects 95% of all children by the age of five years, and infection rates decrease dramatically towards late childhood and adolescence. RV is the leading cause of severe dehydrating diarrhea in children worldwide [1], killing around 600,000 children annually, including 65,000 from the East Mediterranean countries [2]. Consequently, the World Health Organization has recommended RV vaccines as the most effective strategy to prevent RV-related morbidity and mortality [3].

In Saudi Arabia, the annual incidence of deaths from diarrhea in children under five years of age was estimated at 1530, including 474 deaths from RV infection [4]. These data alarmed healthcare providers and decision makers, who introduced the RV vaccine into Saudi Arabia’s national immunization program in 2013 as a two-dose schedule at two and four months of age. Accurate projections of the benefits of the vaccine program in the years following its introduction in Saudi Arabia will require reliable, current data on the disease burden. Over the past 30 years, more than 20 studies have been published in an attempt to characterize the epidemiology of RV diarrhea in Saudi Arabia [5-11], however, only three studies were conducted in the last decade [8-10] which emphasizes the need for up-to-date and comprehensive studies. Furthermore, only one prospective study has been published in children younger than five years [10], and none of the previously published studies addressed the financial burden of RV infections. Because of significant variations in study design, methodologies, study durations, population characteristics, and other factors, heterogeneity exists in detection rates and seasonality for RV infection in Saudi Arabia. The availability of reliable data from properly conducted prospective epidemiological studies in several major cities targeting children under five years, extending for an adequate duration, is vital to assess the burden of RV infections in Saudi Arabia in the pre-vaccination era to allow health care providers and policymakers in the ministry of health to reliably assess the effectiveness of the recently introduced RV vaccines program. Also, such data are vital to increasing the knowledge and awareness of RV infection among physicians and the community.

The objectives of our prospective study were to provide more recent data on the clinical and epidemiological characteristics of RV diarrhea in the pre-vaccine era among young children admitted to a major hospital in Riyadh City, the capital of Saudi Arabia, and to examine the economic burden of RV diarrhea. 

## Materials and methods

Study setting and design

This prospective study was conducted over a 12-month period from January 2012 to December 2012, at King Fahad Medical City, a tertiary healthcare center located in the center of Riyadh, Saudi Arabia. The children’s specialized hospital has a capacity of 240 beds with an annual admission rate of over 3,500 children. Riyadh, the capital, is a large city in Saudi Arabia composed of a mixture of all populations of the kingdom of Saudi Arabia with an estimated population of six million inhabitants. Riyadh is representative of the whole Saudi population because it has the highest rate of immigration from different parts of the country and retains very similar demographic, ethnic, genetic, and dietary characteristics to those of the wider population of Saudi Arabia. In addition, all socio-economic levels are well-represented in Riyadh including lower, middle, and upper-class people. Riyadh experiences a dry desert climate that is hot during the summer and cold in the winter. The pediatric emergency room in the children’s specialized hospital at King Fahad Medical City is accessible to emergency cases of all citizens.

Study population

All children younger than five years of age with acute gastroenteritis presenting to the pediatric emergency department within 21 days of onset and admitted to the hospital were eligible for the study. The criteria for hospital admission of a case of acute gastroenteritis included severe dehydration or moderate dehydration that needed rehydration with intravenous fluid due to the failure of oral rehydration or associated with electrolyte disturbances. Acute gastroenteritis was defined as three or more loose stools per day. Children with other acute comorbidities or chronic diarrhea (lasting > 21 days) were excluded. Dehydration severity was classified in accordance with the European Society of Pediatric Gastroenterology, Hepatology, and Nutrition [12] in three categories: mild (body weight loss < 5%), moderate (5% to 10%), and severe (> 10%). Data collected from each child included age, gender, associated symptoms (like vomiting, fever, and diarrhea), vital signs, degree of dehydration, laboratory tests (including electrolyte and bicarbonate levels), length of hospital stay, and total financial cost of the hospital stay (including cost of the bed, intravenous fluids, and laboratory tests). Metabolic acidosis was defined as bicarbonate levels ≤ 18 meq/L.

Sample collection and testing

Stool samples were obtained from each enrolled child within 24 hours of admission to the hospital and tested for the presence of RV using an enzyme immunoassay. Fecal samples were prepared by adding 0.2 mL of feces to 2 mL of dilution buffer in centrifuge tubes. After thorough mixing, the tubes were incubated at room temperature for 10 minutes. Samples were then spun in a centrifuge for 10 minutes, and the supernatant was separated. Fifty micromoles of the supernatant were tested by enzyme-linked immunosorbent assay using an RV kit following manufacturer's instructions.

Ethical consideration

The institutional review board committee reviewed and approved the study protocol (11-106). The parents of the children participating in the study provided signed informed consent.

Statistical analyses

Data were analyzed using Statistical Package for the Social Sciences for Windows (SPSS; Version 21, IBM, Armonk, NY). Descriptive statistics (frequencies and percentages) were used to describe the categorical study and outcome variables. Pearson’s chi-square test and odds ratios were used to observe and measure the association between the categorical study and outcome variables. Binary multivariate logistic regression was used to identify the independent factors associated with the dichotomous outcome variable (RV-positive and RV-negative). A P-value of < 0.05 and 95% of confidence intervals were used to report the statistical significance and precision of the estimates.

## Results

Clinical characteristics

**Figure 1:**
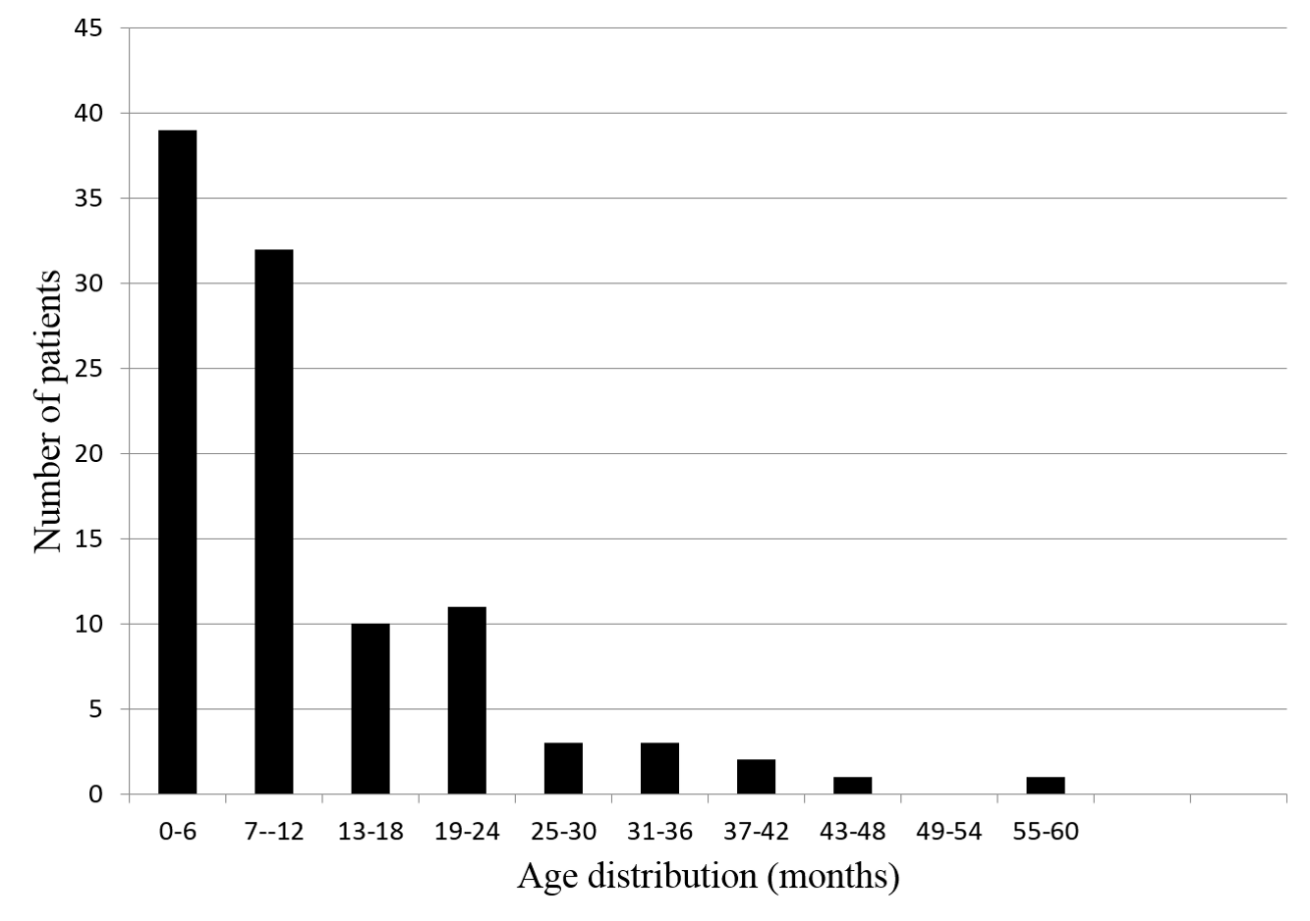
Age distribution of rotavirus diarrhea cases among children under five years of age admitted to the hospital.

The study included 204 children, and their mean age was 9.8 months (± 10.2 months). Of these, 124 were males, and 80 were females. A total of 102 (50%) children were RV-positive. Figure 1 shows the age distribution of RV diarrhea cases among children under age of five admitted to the hospital. Two-thirds (69.6%) were under one year of age, and there was a dramatic decline in the incidence of RV-positive diarrhea after the age of one. Of 39 RV-positive infants under six months old, 11 were two months old or younger (28%).

Table 1 shows the clinical and laboratory characteristics of the 204 children and compares RV-positive diarrhea to RV-negative diarrhea. RV-positive diarrhea was more severe than RV-negative diarrhea as indicated by a higher frequency of vomiting (81% versus 60.7%, P-value = 0.001), significantly lower bicarbonate levels (68.6% versus 31.3%, P-value < 0.0001), a higher frequency of severe dehydration (11.7% versus 3%, P-value = 0.015), and longer hospital stays. Consequent to the longer hospital stay in the RV-positive group, the median financial cost of care in this group was significantly higher than in the RV-negative group (1692 USD versus 1287 USD; P-value = 0.001).

**Table 1:** Clinical and laboratory characteristics of rotavirus-positive cases compared to rotavirus-negative cases.

Variable	RV+ diarrhea, N= 102	RV- diarrhea, N= 102	P-value	Odds ratio (95% CI)
Age (months) ≤ 12 months (%) > 12 months (%)	71 (69.6), 32 (31.3)	70 (68.6), 34 (33.3)	0.80	1.08 (0.60, 1.93)
Gender: male/female (%)	68 (66.6)/35 (34.3)	56 (55)/49 (48)	0.06	1.70 (0.97, 2.97)
Fever (%)	83 (81.3)	71 (69.6)	0.03	1.95 (1.04, 3.65)
Tachycardia (%)	52 (51)	39 (38.2)	0.053	1.72 (0.99, 2.98)
Vomiting (%)	83 (81.3)	62 (60.7)	0.001	2.80 (1.52, 5.19)
Severe dehydration (%)	12 (11.7)	3 (3)	0.015	4.43 (1.21, 16.2)
Hypernatremia (%) (serum Na^+^ > 150mmol/L)	19 (18.6)	9 (8.8)	0.066	2.1 (0.89, 5.0)
Hyperkalemia (%) (serum K^+^ > 5mmo/L)	25 (24.5)	10 (9.6)	0.016	3.1 (1.39, 6.98)
Metabolic acidosis (%)	70 (68.6)	32 (31.3)	<0.0001	4.75 (2.59, 8.72)
Length of stay > 4 days (%)	59 (56.7)	40 (37.7)	0.006	2.08 (1.1, 4.01)
Financial cost in US dollars (median)	1692	1287	0.001	-

Seasonal variation

**Figure 2:**
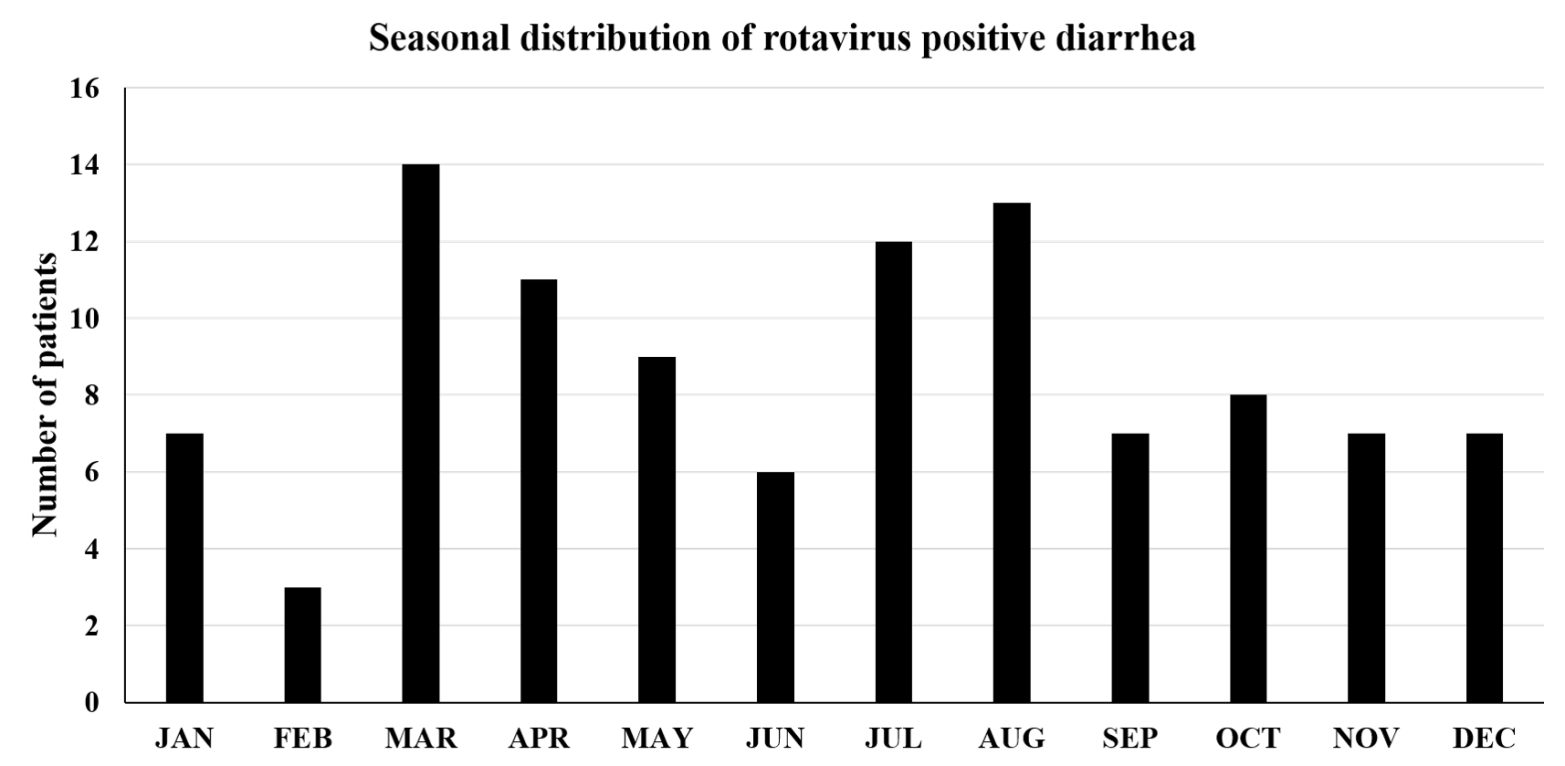
Temporal distribution of rotavirus diarrhea cases among children under five years of age admitted to the hospital during 2012.

Figure 2 shows the temporal distribution of RV diarrhea cases among children under five years old admitted to the hospital during 2012. Admissions due to RV-positive diarrhea were observed throughout the year, with a median of 7.5 cases per month, but with more cases observed during the spring and the summer, accounting for 64% of the total admissions.

## Discussion

The findings from our study confirmed that RV, prior to the launch of the RV vaccine, was still a major cause of hospitalization for acute gastroenteritis among children under five years of age, accounting for 50% of cases. Several RV-associated pediatric diarrhea studies had been performed in other cities in Saudi Arabia. However, most studies only focused on the epidemiological features. The present study not only provided an updated picture of the epidemiological features of RV infections prior to inclusion of the RV vaccine in the national immunization program in 2013 but also compared the clinical peculiarity and severity of symptoms between RV and non-RV associated gastroenteritis and estimated the economic burden of RV infections. Few of the available studies are recent enough to have a meaningful bearing on the current situation in Saudi Arabia, as most studies cover the period from 1982 to 1996, and very few studies were conducted after 2003 [8-10]. In addition, comprehensive knowledge of RV infection epidemiology in Saudi Arabia is limited by several factors, the most prominent of which are the limited sample size, the variability in study duration (< 12 months), recruitment of different age groups including adults, variable study setting (mixing inpatients and outpatients), and retrospective nature of the these studies [5]; only one prospective study had been conducted to date [13]. The present prospective study has addressed many of these limitations to provide up-to-date data needed prior to vaccine introduction.

Earlier epidemiological studies of RV-related diarrhea in Saudi Arabia suggested that RV was the most common cause of hospitalization for diarrhea among children under five years of age, ranging from 40% in the 1980s [14-15] to 65% in 2010 [9]. RV infection occurred mostly throughout the year with a few studies showing peaks during the winter [8, 15] or summer months [14, 16]. RV-related diarrhea was largely limited to children between six and 24 months of age. In our study, unlike previous studies, most admissions for acute RV gastroenteritis were for infants (approximately 70%), with 55% of admitted patients under six months old. The predominance of RV infections during infancy in our study has also been observed in another study conducted during the same period [9]. This observation could be attributed to the increasing practice over the past decade of feeding young infants milk formula instead of breast milk [17]. Maternal anti-RV antibodies acquired through breastfeeding and the high concentration of immunoglobulin A in breast milk protect infants from RV gastroenteritis.

The predominance of RV infections during infancy in our study provides scientific support for the recent introduction of RV vaccine into Saudi Arabia’s national immunization program, as a two-dose schedule early in life at two and four months of age to assure a higher effectiveness of the vaccine in the country. In further analysis of the data, we observed that 11 RV gastroenteritis cases of the total 102 (11%) were two months old or younger. Since the first vaccine dose is currently given to infants at two months of age, infants younger than this age will not be protected by the vaccine and will remain susceptible to RV infection. Therefore, future studies in the era since the RV vaccine was made available are needed to examine if young infants are susceptible to RV infection and whether the first RV vaccine dose needs to be given at one month of age.

RV infections impose a heavy economic burden due to direct costs (e.g., consultation, emergency, hospitalization, medications) and indirect costs (e.g., parents’ working days absence and additional use of diapers). Data from our region on the burden of RV gastroenteritis regarding mortality, morbidity, and economic burden is lacking. To our knowledge, our report is the first in Saudi Arabia to report on the economic burden of RV infection. We did not have the resources to conduct surveillance for cases of moderate or mild diarrhea treated in outpatient clinics or at home. Therefore, the economic burden of RV gastroenteritis cases admitted to our hospital during the study period is indeed an underestimation of the tremendous economic burden imposed by RV cases in the community. However, our data generated baseline information on RV infection burden, which could be used as a reference for the interpretation of post-vaccine RV disease trends [18-19].

Our study has several limitations. The data presented are from a single tertiary care center in Riyadh, which may not be representative of the entire country, and therefore, the findings from the present study may not be generalized. Furthermore, we did not have the resources to perform genotyping of RV strains in the years prior to the introduction of the RV vaccine in Saudi Arabia; such data can be useful to plan effective new RV vaccines.

## Conclusions

In conclusion, our study shows a high prevalence of RV infections among infants admitted to the hospital for acute gastroenteritis. Furthermore, RV infection is associated with severe diarrhea and significant financial burden.
